# Statistical Reports on the Health of the Navy, for the Years 1830—6, Inclusive. Part II

**Published:** 1842-04-01

**Authors:** 


### Statistical Reports on the Health of the Navy, for the Years 1830—6 inclusive.—Part II. (Cape of Good Hope—West Coast of Africa—East Indies—Home and various Forces.) Together with the Totals for Seven Years throughout the Service. Folio, pp. 339. 1841.

This Report is not less valuable or interesting than its predecessor, of which we gave a full account at the time. The Reporter is entitled to the greatest praise for the vast labour which he has expended on these returns, and the clear judgment which he has evinced in their arrangement, as well as in the numerous remarks and reflections interspersed through his pages. These statistical reports compel us to unlearn a great deal of what we thought to be Gospel heretofore—and to admit as truths many statements which a few years ago we would have treated as fictions. Thus the comparative salubrity of the home and of foreign stations upsets many of our favourite dogmas and doctrines, so long cherished as unerring axioms!

"Notwithstanding the remarks which have already been made on the sanatory influence of the South American climate, it may be permitted to glance at it again, on account of its singular and unexplained power, at least in its operation on board ship, to which alone these observations apply. Compared with the climate of the British channels and ports, it appears, drawing conclusions according to prevalent hypotheses and accepted dogmata, to have almost every thing against it. Most of it is within the tropics. A great portion of its shores is still in a state of nature; all of it is teeming with vegetable and animal productions growing, or decomposing, rapidly. Rain falls in torrents at intervals in many places; and evaporation, atmospheric heat being intense, proceeds rapidly. High winds are rare; calms are common. Yet with all these apparent elements of disease and destruction, the mortality of the squadron employed there from 1830 to 1836, was less than in the force employed at home during the same period. Compared with other tropical positions, particularly the coast of Africa and the West Indies, nothing appears in its favour; compared with the latter, something in the natural condition of the soil and its superabundant products, appears against it; yet its mortality is not one-third part so great. These things, and others of similar import which might be cited, show the ignorance which generally prevails on the subject of climate as affecting health; and that much must be unlearned, as well as learned, before any thing deserving the name of knowledge shall be obtained on this very interesting subject." v.

It is curious to compare the statement with the frightful mortality by scurvy on the same station, in the days of Anson. It has been confidently and currently believed that the disasters of the Centurion were owing to the want of lemon-juice—an error which Dr. Johnson long ago pointed out in his work on Tropical Climates, and which, we rejoice to see repudiated by the able reporter of the work before us.

"It may be affirmed safely, because much observation, and the inherent nature of things,—the necessary relation of cause and effect,—bear testimony to its truth, that, if abundance of wholesome food, pure water, ample clothing, cleanliness and ventilation, as they now are, had at that time been enjoyed in the navy, without any aid from citric acid, the tremendous mortality then resulting from scurvy, which half depopulated the ship, nearly frustrated the objects of the expedition, and was, in some measure, an imputation on the character of the country, might have been spared." v.

Dividing the last 60 years into three periods, we find the ratio of mortality in the navy generally to be as follows: viz. in 1779, there was one death in every eight men employed, annually. In 1811, it was 1 in 32. In the period embraced by the present report (1830—6) the ratio of mortality was 1 in 72!!

On reading this, our first sensation is that of surprise—and next, of admiration at the wonderful pitch of improvement to which the economy of the navy has arrived—in discipline, food, clothing, ventilation, and every thing that concerns the health and comfort of our gallant tars! It must be remembered, however, that, during the seven years embraced in this report, we were at peace, and the seamen were volunteers—which makes a vast difference in the comparative health of this and the other periods.

### I. Cape of Good Hope and Coast of Africa.

1830.

The Cape station includes the eastern as well as the western coasts of that huge triangular peninsula, having the Mediterranean for its base, and the Cape itself for its apex. Over a great deal of this extended line of coast there is considerable similarity of climate—at least as far as temperature is concerned. Almost the whole is intertropical—but varying greatly in point of salubrity. This coast is infinitely more unhealthy than the Mauritius, which lies also within the tropics, but has the advantage of being an island, and at a great distance from the African main. That island, indeed, suffered severely from epidemic cholera; but this is no criterion of general salubrity or otherwise.

The mean force of 1830 was 1,578—number of vessels 23, mostly small, there being only two frigates in the whole. The principal duty was the prevention of the embarkation of slaves, and the pursuit of suspected vessels afterwards. This duty is a very harassing one, and attended with much anxiety and fatigue, as well as privations—circumstances, co-operating with an essentially unhealthy climate, which occasion great sickness and mortality. In the above year, 2279 men were put on the sick-list—being about the ratio of 14 to 10 of the crews. Thus some men must have been several times on the sick-list, whilst others were not at all so. Of the above number 57 died—being at the rate of about 36 in the thousand. There were also 104 invalided—or 66 in the thousand.

Great as was this rate of sickness and mortality, it was by no means so much as in some other years. The fevers, which are the grand causes of destruction on this station, come round, like Sir Robert Peel's bad seasons, in cycles, though very irregularly. The statistics, therefore, of one, two, or three years may be often found totally inadequate to the formation of a correct estimate of the mean ratio of sickness on the African, and indeed on many other stations. Of the 57 deaths in 1830, 41 were from fever. There were 250 cases in all—20 "ephemeral"—169 continued fever—45 remittent—and 35 intermittent fever. There is every reason to believe that all of the cases denominated "continued" fevers, were, in reality, remittents, the remissions being obscure.

In the "PLUMPER," with a complement of 50 men, there were 35 cases of fever, of which 24 died—a mortality so frightful, that it gives a melancholy idea of African fever, which, on this occasion, equalled the late ravages of disease in the Niger! The island of Fernando Po was found more destructive to life than even the adjacent continent, and has been abandoned as a settlement.

After fever, the bowel complaints were the most formidable enemies of the European constitution. These included diarrhœa, dysentery, enteritis—"all closely associated affections, one often running into the other so insidiously, and by movements so difficult to observe, that it was impossible sometimes to decide the exact limits of each, though there was great difference between their extreme points—diarrhœa being often the most trivial, and acute dysentery one of the most dangerous affections to which seamen are subject."

Of 66 cases of dysentery, 4 ended fatally, independent of two deaths from "inflammation of the bowels." There were also six invalided, and nine sent to hospital, where three of the four deaths occurred. The dysenteric affections occurred almost entirely on the Mauritius station—the fevers on the African coast.

There were 27 cases of pulmonary inflammation, all of which were cured on board, except two cases which, becoming chronic, were sent to England. The rheumatic cases amounted to 75, seven of which were invalided. There were seven cases of phthisis—one of which terminated fatally at sea—two were sent to hospitals—and three were invalided—leaving one under treatment at the end of the year. Though these cases are comparatively rare on the Cape station, they run their course in a most rapid manner—shewing the absurdity of sending consumptive patients to warm climates.

Thirty cases of liver disease were entered on the sick-list, including acute and chronic inflammation. Two of these died in hospital. One hundred and twenty cases of "primary venereal disease"—71 of syphilis, and 49 of gonorrhœa. Wounds, accidents, and ulcers were not formidable, though tolerably numerous.

1831.

The mean force employed, this year, was 1706—and the number of cases entered on the sick-list 2337—or 1369 for every 1000 men. Of these, 73 were sent to hospitals—82 were invalided—and 35 died, or $20\frac{1}{2}$ out of every 1000 men.

It is evident that the mortality this year, as compared with the preceding year, was not great.

"The vessels on the West coast of Africa were employed in counteracting the operations of slave traders, in preventing the embarkation of slaves, or in following, and endeavouring to seize the vessels in which they were carried. For the first purpose they must keep close to the shore, particularly at the mouths of rivers, in creeks, and other confined situations, abounding in the cause of violent fever. For the second, they continue long and anxiously employed at sea, in low latitudes, exposed to high degrees of heat, with heavy periodic rains, living on salted food, much exposed in boats, and occasionally coming into collision with the slave ships. Such service is, as would be expected, prejudicial to health, often highly destructive of life; yet there are years in which the destructive power is not exerted. Such exceptions, often extending over considerable spaces of time, occur in this and other unhealthy localities, perplexing, and too often discouraging the inquirer. The cause would appear to be evolved as abundantly as at other times, yet the effect does not follow. This apparent want of correspondence between cause and effect is apt to make the man who is endeavouring to trace and satisfactorily exhibit the entire chain of morbific and morbid action, the febrific cause and the febrile effects,—the first emanating from the spot where the last is excited—despair of obtaining his object, and therefore to abandon it; but it is hoped that the time is not far distant, when, a right method of investigation being adopted, and vigorously pursued, with necessary qualifications for conducting it properly, this interesting and important problem shall be solved." 10.

1832.

The sickness and mortality this year were intermediate between 1830 and 1831. Less than in the former year—greater than in the latter. There is nothing very particular in the statistical report of this period.

1833.

The mean force this year was 1357—and 1817 cases of illness were recorded—a ratio of 1338 per thousand men. The ratio of mortality was 25 deaths in every thousand men—or 34 in the whole—almost exactly the same rate as in 1832.

1834.

Mean strength was 1782, and 1493 cases of sickness. Of these, 33 died, or 27½ per thousand.

1835.

Mean strength employed 1497 men—and 2126 cases of illness. Of these, 24 died—a proportion of 16 in the thousand men, and 38 were invalided. This, therefore, has been the most favourable year of any yet considered—not only in the rate of mortality, but also in that of invaliding—being less than half of the mortality in 1830. It is impossible, however desirable, to account for these fluctuations in *effects*, while the causes remain apparently the same.

"The mode of life, of diet, drink, clothing, sleeping and watching, were the same; the nature and the places of service were the same. It would be highly interesting to ascertain why the indigenous cause of fever,—for on that the difference depends, was in such different degrees of force, though always existing, in these years. It has evidently periods of increasing, maximum, and decreasing, force; this it has in common, perhaps, with the cause of other febrile endemics, at least those of hot regions." 27.

1836.

The mean strength was 1355—number of sick cases 1347. Of these, 40 were invalided, or about 3 per cent.—and 20 died, or about 1½ per cent. of the whole force. There is but little difference between this year and the last—what difference there is, is in favour of 1836.

Aggregate of the Seven Years.

The whole force employed, was 10591 men, and the total number of deaths, both on the station and at the hospitals at home, were 263, or 2½ per cent. per annum. The wonder is, that the mortality on a station including the West coast of Africa, should be so moderate. Thus in the boasted climate of Naples, the mortality annually is at least 35 per thousand inhabitants, or $3\frac{1}{2}$ per cent.—one per cent. more than among our seamen on the coast of Africa!! This appears astounding, at first sight; but, when we reflect that in Naples, the whole population is included—old and young, sick and sound—whereas, our seamen are generally in the prime of life—the wonder is much abated, though it does not entirely vanish.

But the loss on the station in question, though apparently much smaller than might have been expected, was yet three times as much as on the South American—as 25 to 9, compared with the Mediterranean—and as 12 to 9, compared with the West Indies.

The period of seven years on the Cape station, as above analyzed, presented a very favourable specimen. If the seven preceding, and the seven succeeding years were included in the survey, the picture would be many degrees darker than it is. The fact is, that twenty years are scarcely sufficient to strike an average of sickness and mortality on this, and perhaps on any other station between the tropics—or even beyond them.

The number of tables constructed for the septenniad above-mentioned is very great; but these we cannot notice in this brief abstract. They are highly creditable to the labour of Dr. Wilson and the able Physician-General of the Navy. The late period of the quarter, at which we received the volume, prevents us from noticing the other commands and stations comprised in the Report till next number.

